# Analysis of the CDK4/6 Cell Cycle Pathway in Leiomyosarcomas as a Potential Target for Inhibition by Palbociclib

**DOI:** 10.1155/2019/3914232

**Published:** 2019-01-21

**Authors:** Michael J. Böhm, Ralf Marienfeld, Daniela Jäger, Kevin Mellert, Adrian von Witzleben, Silke Brüderlein, Mathias Wittau, Alexandra von Baer, Markus Schultheiss, Regine Mayer-Steinacker, Frank G. Rücker, Peter Möller, Lars Bullinger, Thomas F. E. Barth

**Affiliations:** ^1^Institute of Pathology, Ulm University, Ulm, Germany; ^2^Department of General and Visceral Surgery, Ulm University, Ulm, Germany; ^3^Department of Trauma Surgery, Ulm University, Ulm, Germany; ^4^Department of Internal Medicine III, Ulm University, Ulm, Germany; ^5^Medical Department, Division of Hematology, Oncology and Tumor Immunology, Charité, Berlin, Germany

## Abstract

Leiomyosarcoma (LMS) is characterized by high genomic complexity, and to date, no specific targeted therapy is available. In a genome-wide approach, we profiled genomic aberrations in a small cohort of eight primary tumours, two relapses, and eight metastases across nine different patients. We identified CDK4 amplification as a recurrent alteration in 5 out of 18 samples (27.8%). It has been previously shown that the LMS cell line SK-LMS-1 has a defect in the p16 pathway and that this cell line can be inhibited by the CDK4 and CDK6 inhibitor palbociclib. For SK-LMS-1 we confirm and for SK-UT-1 we show that both LMS cell lines express CDK4 and that, in addition, strong CDK6 expression is seen in SK-LMS-1, whereas Rb was expressed in SK-LMS-1 but not in SK-UT-1. We confirm that inhibition of SK-LMS-1 with palbociclib led to a strong decrease in protein levels of Phospho-Rb (Ser780), a decreased cell proliferation, and G_0_/G_1_-phase arrest with decreased S/G_2_ fractions. SK-UT-1 did not respond to palbociclib inhibition. To compare these *in vitro* findings with patient tissue samples, a p16, CDK4, CDK6, and p-Rb immunohistochemical staining assay of a large LMS cohort (*n*=99 patients with 159 samples) was performed assigning a potential responder phenotype to each patient, which we identified in 29 out of 99 (29.3%) patients. Taken together, these data show that CDK4/6 inhibitors may offer a new option for targeted therapy in a subset of LMS patients.

## 1. Introduction

Leiomyosarcomas (LMS) are defined by smooth muscle differentiation and are characterized by complex and highly variable genetic abnormalities [1–3]. They account for as many as 24% of all soft tissue sarcomas, and the yearly incidence is estimated at around 1.23/100,000 [4]. The pathogenesis and genetic complexity of LMS are still poorly understood [3].

Clinically, radical surgery remains the cornerstone of treatment in local LMS disease, supported by adjuvant chemotherapy and radiotherapy [5]. Nevertheless, local recurrences are common in up to 39%, and LMS may metastasize in up to 81% of cases [6, 7]. Chemotherapy regimens are currently based on doxorubicin/ifosfamide or docetaxel/gemcitabine [8, 9], and new protocols approved for treatment include substances such as trabectedin and pazopanib [10–12]; but no effective targeted therapies are available to date. The 5-year overall survival in advanced stages still remains poor, and median overall survival ranges between 45 and 92 months [13–15].

Amplification and overexpression of *CDK4* have been reported in LMS in the past [16], and other events such as *RB1* deletions and *CDKN2A* promoter hypermethylation have been described [17, 18]. Specific CDK4 and CDK6 inhibitors, such as palbociclib, offer a new potential target in the underlying p16-CDK4/6-Rb pathway (p16 pathway) since palbociclib has been approved for treatment of breast cancer and showed favourable outcomes in Phase I-II clinical trials in various types of cancer, such as mantle cell lymphoma, multiple myeloma, liposarcoma, melanoma, and germ cell tumours [19–25]. In mice, a favourable effect on leiomyosarcoma by CDK4 inhibition was observed [26]. Furthermore, Francis et al. showed that palbociclib leads to a reversible arrest in the G_1_ phase of the cell cycle and that Rb-positive cell lines like SK-LMS-1 and HT-1080 are more sensitive to agents that work preferentially in the S-G_2_ phase such as doxorubicin and WEE1 kinase inhibitors in xenograft models [27]. However, the underlying mechanism has not been investigated in leiomyosarcoma samples *in situ*. We aimed at dissecting molecular genomics in LMS, confirming the role of the p16 pathway in LMS *in vitro*, and ultimately separating a potential responder phenotype for palbociclib treatment for patients with LMS by including patients with primary LMS, recurrences, and metastases in the analysis.

## 2. Materials and Methods

### 2.1. Leiomyosarcoma Tissue Bank

Paraffin blocks of LMS samples from 99 patients were available from the archive of the Institute of Pathology, University of Ulm, Germany. 24 tumours were of uterine origin versus 75 nonuterine tumours. From these, 20 arose in the retroperitoneum, and 55 were localized in other tissue sites (Supplementary Table S3).

The median age at diagnosis was 60.3 years (range: 27 to 85 years); 39 patients were male and 60 female. Primary tumour size varied between 0.3 and 30 cm; 61 patients had metastatic disease. Follow-up data were available for 98 patients: 33 are alive (median follow-up time: 41.9 months). The diagnosis was based on histological subtyping (WHO 2013) and identified GII/GIII LMS in the majority of cases (GI, *n*=2; GII, *n*=47; GIII, *n*=50) according to the Federation Nationale des Centers de Lutte Contre le Cancer (FNCLCC) grading system. LMS patient samples were pseudonymized to comply with the German law governing the correct usage of archival tissue for clinical research. All experiments were conducted in accordance with the Declaration of Helsinki and the guidelines of the Ethics Committee of the Federal General Medical Council [28], and the study was approved by the local Ethics Committee of the University of Ulm (usage of archived human material 03/2014).

### 2.2. CNV Analysis Using the OncoScan FFPE Express 2.0 Array

This method combines the molecular inversion probe technology with a SNP assay. It provides whole genome coverage with ∼330000 probes and a probe density of 1 probe per 0.5–2 kb for 201 tumour suppressor genes and oncogenes as well as a median backbone of 1 probe per 9 kb. We analysed 18 paraffin LMS samples from 9 different patients. Copy number calls were calculated automatically using the TuScan and SNP-FASST2 algorithm. Calls are based on the software's default threshold, for SNP-FASST2 log_2_-ratios are classified as follows: high copy gain 1.2, one copy gain 0.3, copy loss −0.3, and homozygous copy loss −1.2. For the TuScan algorithm, only the high copy gain calls are adjustable, and the default threshold of 4 total copy numbers was used to make these calls.

### 2.3. *In Vitro* Experiments

For our *in vitro* studies, we used the two commercially available cell lines SK-LMS-1 (courtesy of Karlisch et al., Department of Gynaecology and Obstetrics, Marien Hospital Witten, Witten, Germany) [29] and SK-UT-1 (purchased from CLS Cell Lines Service GmbH, Eppelheim, Germany). For inhibition experiments, the selective CDK4/CDK6 inhibitor palbociclib (PD 0332991) was used. As controls for the Western blot analysis, we used HeLa cells (Leibniz-Institut DSMZ, Bochum, Germany). Genotyping of the cell lines was accomplished using the GenomeLab STR Primer Set Kit (Beckman Coulter, Krefeld, Germany) and the AmpliTaq Gold DNA Polymerase (Life Technologies, Carlsbad, CA).

### 2.4. Western Blot Analysis

The following antibodies were used: CDK4 (1:1000, DCS-31.2, Zytomed Systems, Berlin, Germany, 603–1840), CDK6 (1:1000, Abcam, Cambridge, UK, ab54576), Rb (1 : 2000, 4H1, Cell Signaling Technology, Danvers, MA, 9309), Phospho-Rb (Ser780, 1 : 1000, Cell Signaling Technology, 9307), p16 (1 : 500, JC8, Santa Cruz Biotechnology, Dallas, TX, sc-56330), ERK2 (1 : 2000, C-14, Santa Cruz Biotechnology, sc-154), and *β*-tubulin (1 : 2000, TUB 2.1, Sigma Aldrich, T4026).

### 2.5. Cell Counting

SK-LMS-1 was subcultured and treated with 100 nmol/l and 1000 nmol/l of palbociclib. Plates were randomly divided into 3 different groups, and automatic cell counting of one group was performed every 24 hours for up to 3 days using Vi-CELL (Beckman Coulter). Three independent experiments were performed.

### 2.6. FACS Analysis

In three independent experiments, SK-LMS-1 cell lines were treated as above with 100–1000 nmol/l palbociclib for 24 and 48 hours. Analysis was performed using the FACSCalibur (Becton Dickinson, Franklin Lakes, NJ), and the data were analysed with the CellQuest software (Becton Dickinson).

### 2.7. MTS Cell Proliferation Assay

CellTiter 96 Aqueous One Solution Cell Proliferation Assay from Promega (Fitchburg, MA) was used to determine the cell viability. SK-LMS-1 and SK-UT-1 cells were cultivated in 96-well plates and incubated with increasing concentrations of palbociclib (0–1000 nmol/l) in the medium. After 3 days of incubation at 37°C, MTS was added to the medium, and the absorption was measured at 490 nm using a microtitre plate reader as previously described [30]. MCF7 and CAL51 cells served as positive and negative controls as previously published [31].

### 2.8. FISH Analysis

34 samples from 25 patients were investigated for *CDK4* amplification. A standard fish protocol was used together with the Kreatech *CDK4* (12q13)/SE 12 FISH probe (Leica Biosystems, Wetzlar, Germany, KBI-10725). Across each slide, fluorescence signals from 100 different nuclei were analysed, and the ratio of the number of *CDK4* signals to the number of centromere 12 signals was calculated. ZytoLight SPEC CDKN2A/CEN 9 Dual Color Probe (ZytoVision, Bremerhaven, Germany, Z-2063-50) and Vysis LSI 13 (13q14) SpectrumGreen Probe (Abbott Molecular, Des Plaines, IL, 08L67-020) were used for cell lines SK-UT-1 and SK-LMS-1.

### 2.9. Immunohistology

Tissue sections of 2 *µ*m from representative paraffin blocks were stained. Briefly, for antigen retrieval, the sections were heated in citrate buffer at pH 6 in a steamer for 20 minutes. The primary antibody was used in the below-cited concentrations in phosphate buffer saline (PBS); slides were incubated with 50 *µ*l per section in a humid chamber at room temperature for 30 minutes. As a detection system, we used the EnVision Kit (Dako, Carpinteria, CA, USA) according to the manufacturer's protocols. The proportion of cells showing a positive staining was categorized as follows: “no staining detected” (−); “staining in up to 30%” (+); “staining in more than 30% and up to 70%” (++) and “staining in more than 70%” (+++) of the total number of tumour cells analysed.

The following antibodies were used: CDK4 (1 : 100, Zytomed Systems), CDK6 (1 : 100, Abcam), Phospho-Rb (Ser780; 1:250, Cell Signaling Technology), p16 (1 : 100, Santa Cruz Biotechnology), Ki-67 (1 : 200, clone MIB-1, Dianova, Hamburg, Germany), and cleaved caspase-3 (Asp175, 1 : 1005A1E, Cell Signaling Technology, 9664).

### 2.10. Statistical Analysis

One-way ANOVA was performed using GraphPad Prism Software Version 6 (San Diego, CA). SPSS Statistics 24.0 (IBM, Armonk, NY) was used for clinical follow-up analysis. We performed Kaplan–Meier analysis combined with Mantel–Cox testing, and Pearson correlation coefficient of clinical and pathological parameters was performed. Results with *p* < 0.05 were regarded as statistically significant. Significance levels are indicated in the figures as follows: ^*∗*^=*p* ≤ 0.05, ^*∗∗*^=*p* ≤ 0.01, ^*∗∗∗*^=*p* ≤ 0.001, and ^*∗∗∗∗*^=*p* ≤ 0.0001.

## 3. Results

The 18 samples analysed comprise tumour sites from nine different patients (three uterine versus six nonuterine). From these, seven had advanced disease (two relapses and five metastases), while the remaining two patients had locally controlled tumours at the time of analysis (Supplementary Table S2).

The copy number variation (CNV) analysis demonstrated a large amount of genetic alterations across most cases. The average of copy number calls/sample across all samples was as follows: 243 total aberrations, 74 copy losses, 132 copy gains, 33 high copy gains, and 4 biallelic copy losses.

Primary tumours (*n*=8) had an average of 263 total aberrations/sample with 88 copy losses, 139 copy gains, 30 high copy gains, and 7 biallelic copy losses. Metastases (*n*=8) had an average of 227 total aberrations with 64 copy losses, 121 copy gains, 40 high copy gains, and 2 biallelic copy losses. Recurrences (*n*=2) had an average of 224 total aberrations, 56 copy losses, 149 copy gains, 17 high copy gains, and 2 biallelic copy losses.

Comparison of uterine versus nonuterine tumours showed the following: Uterine tumours (*n*=7) had an average of 220 total aberrations/sample with 68 copy losses, 127 copy gains, 23 high copy gains, and 2 biallelic copy losses. Nonuterine tumours (*n*=11) 258 total aberrations/sample with 77 copy losses 135 copy gains, 40 high copy gains, and 6 biallelic copy losses. Recurrent regions (cutoff <25%, *p* value <0.05) with high copy gains were identified on chromosomes 12 and 17 (Table 1).

**Figure 1:**
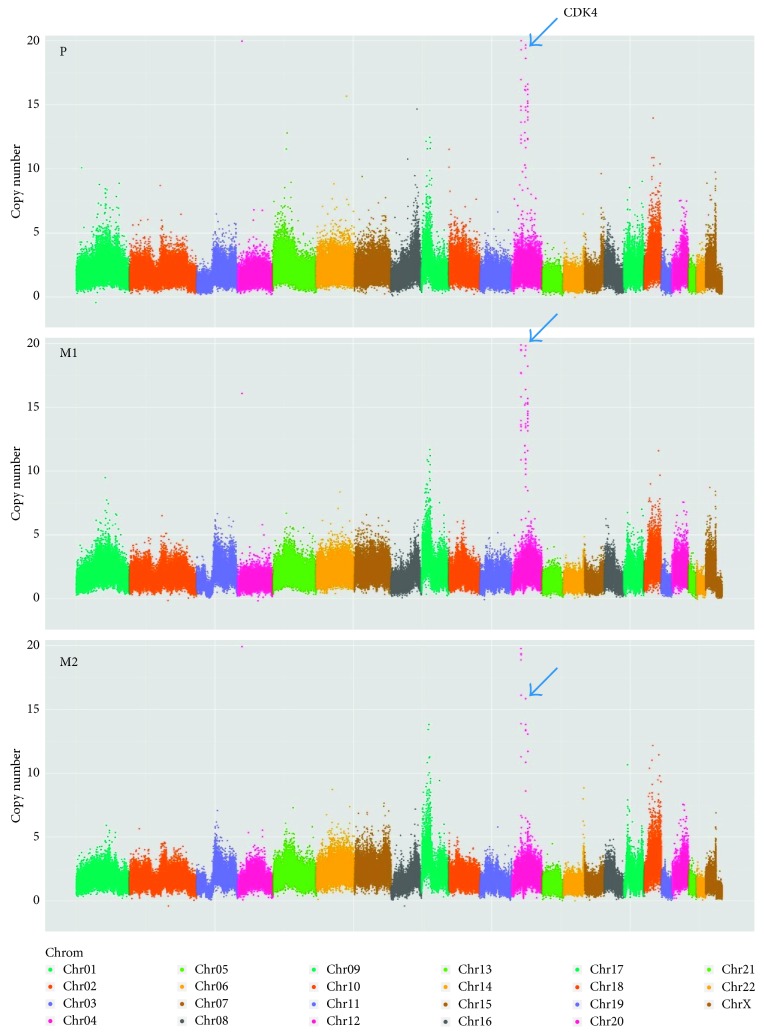
Comparison of copy number plots from three tumour samples of patient #59. The genome is represented on the *x*-axis and chromosomes are delineated by changing colours starting with chromosome 1 on the left. A persistent CDK4 amplification in all three samples is shown. P, primary tumour; M1, metastasis #1; M2: metastasis #2.

The region on 12q14.1 involved the *CDK4* locus, which was amplified in 5/18 (27.8%) samples, corresponding to 2/9 (22.2%) patients (Figure 1). In the second step, we investigated this finding in a larger cohort; to this end, FISH analysis was performed with a *CDK4* specific probe. FISH analysis of *CDK4* showed amplifications in 4 out of 25 patients (14.7%).

To analyse the role of the pathway, we performed Western blot analysis of the two cell lines SK-LMS-1 and SK-UT-1. The origin of the cell lines was confirmed by STR results (Supplementary Table S4). As shown in Supplementary Figure S1A, SK-LMS-1 expressed CDK4, CDK6, and Rb, while p16 protein was absent. SK-UT-1 showed CDK4 expression, but showed only very weak Rb protein and strong p16 expression. Therefore, SK-LMS-1 was selected as the *in vitro* model for palbociclib inhibition.

For pathway analysis, we performed Western blot analysis of SK-LMS-1 after incubation with palbociclib. After inhibition with various concentrations of palbociclib for up to 2 days, we detected a clear decrease in p-Rb (Ser780) and persistent Rb-expression with increasing palbociclib concentrations (Supplementary Figure S1B). These findings suggest that palbociclib blocks the pathway efficiently.

In another approach, we treated SK-LMS-1 cells as described above and performed cell cycle analysis by FACS. Supplementary Figure S2A shows an increase in the G_1_ phase and decrease in the S and G_2_ phase. The sub-G_1_ phase was only slightly increased, and no apoptotic cells were detected by cleaved caspase-3 staining (Supplementary Figure S3B). By direct cultivation of SK-LMS-1 cells on glass slides, we studied the effect of palbociclib on cell morphology. As shown in Supplementary Figure 3C, there were populations of enlarged multinucleate cells, which is a morphological change observed in senescent cells. We further investigated the effect of palbociclib on cell growth and therefore examined the effect of a 3-day treatment of 100 nmol/l and 1000 nmol/l by cell counting. Supplementary Figure S2B shows that treatment leads to decreased proliferation when comparing treated with untreated cells, and this effect was concentration dependent. These findings are consistent with our immunocytological data, since we observed a decreased Ki-67 staining in treated cells (Supplementary Figure S3A).

**Figure 2:**
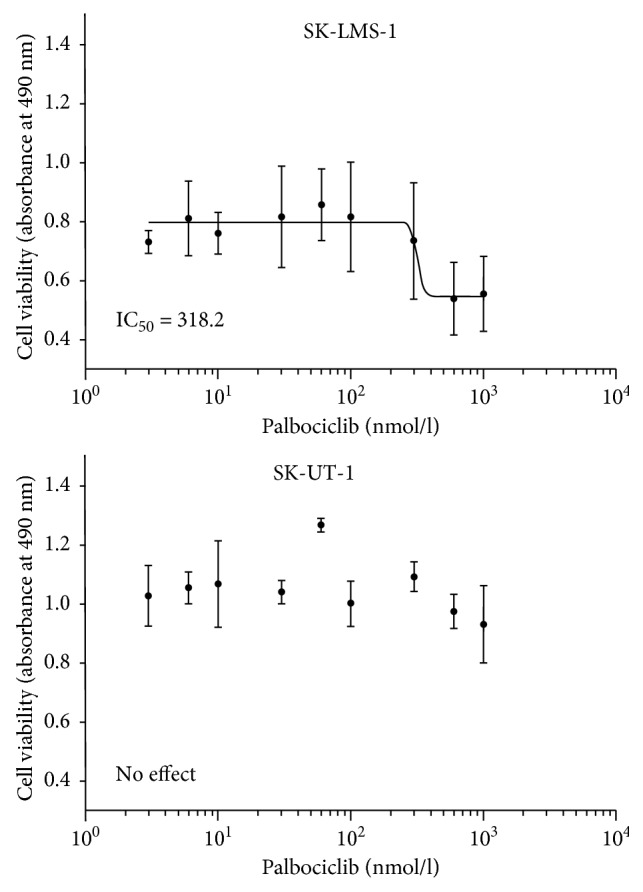
Growth inhibitory effect of palbociclib on SK-LMS-1 and SK-UT-1. Cells were cultivated on 96 plates and treated with increasing concentrations of palbociclib (3–1000 nmol/l) for 72 hours. The MTS assay was used for determining cell viability.

For SK-LMS-1, the IC_50_ value of palbociclib was 318.2 nmol/l. The incubation of SK-UT-1 with palbociclib had no effect (Figure 2). FISH analysis of SK-LMS-1 showed a relative loss of 70% of the *CDKN2A* region, an amplification of *CDK4* with 5 copies, and 2 *RB1* gene copies. In SK-UT-1, we saw a normal *CDKN2A* constellation and 2 copies of *CDK4* and a single copy deletion for *RB1*.

In our OncoScan patient group, gene amplification is accompanied by overexpression for CDK4 (4 samples) and CDK6 (2 samples). Biallelic loss of *RB1* and *CDKN2A* each translates into loss on the protein level in one sample confirming the assumed gene dosage effect on the protein axis (Supplementary Table S1).

**Figure 3:**
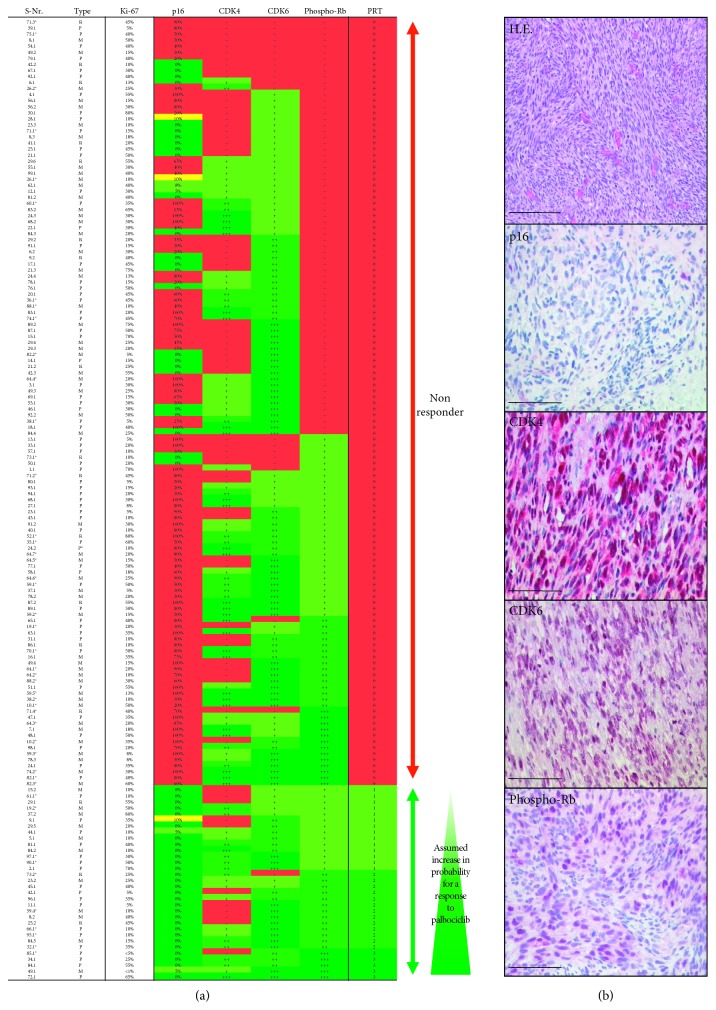
(a) Expression of p16 pathway proteins and Ki-67 index of leiomyosarcoma tissue and assumed increased probability to potential response of palbociclib treatment. Multiple samples from the same patient are marked by their matching sample number (e.g., 6.1 and 6.2); uterine tumours are marked with ^*∗*^. S.-Nr., sample number; PRT, potential responder phenotype; P, primary tumour; M, metastasis; R, recurrence; and P^*∗*^, primary tumour after neoadjuvant chemotherapy. (b) Exemplary H.E. staining and immunohistochemistry. Negative staining for p16, strong expression of CDK4, CDK6, and p-Rb. Bars: 100 *µ*m and 200 *µ*m for H.E.

To compare our *in vitro* findings with patient tissue samples, an immunohistochemical staining for p16, CDK4, CDK6, and p-Rb (Ser780) of each sample was performed for a larger cohort of 99 patients with 159 tumour samples. Each sample of our cohort was scored as described above with regard to expression of p16, CDK4, CDK6, and p-Rb (S780). From 159 examined samples, 92 (57.8%) were positive for CDK4, 138 (86.8%) for CDK6, and 90 (56.6%) for p-Rb. p16 was negative in 54 (34.0%) samples and below ≤10% in 7 (4.4%) samples. On the basis of our *in vitro* results, we defined four types of LMS by scoring the expression profiles from 0 to 3, and potential responder types were classified as described previously [31]. Therefore, the potential nonresponder type (type 0) was defined by either p16 expression in over 10% of the tumour cells, lack of the p-Rb molecule, or the absence of both CDK4 and CDK6. The best assumed responder profile is characterized by expression of p-Rb (S780) in more than 70% of cells (+++). The other categories describe immunoreactivity for p-Rb (S780) in increasing percentages of positive cells: 0 = p16 > 10% positive; p-Rb negative; CDK4 and CDK6 negative; 1 = p-Rb is +; 2 = p-Rb is ++; 3 = p-Rb is +++. Applying the above results to our cohort, we identified 32 out of 159 samples (20.1%) as potential responders (Figure 3). Using the above profile response, profile samples were stratified as follows: 14 (type 1), 13 (type 2), and 5 (type 3). Further stratified by tumour site, we report that primary tumours (*n*=80) were positive for CDK4 in 60.0%, for CDK6 in 85.0%, for p-Rb in 60.0%, and ≤10% for p16 in 37.5%. Therefore, 22.5% (18 samples) of primary tumours qualified as potential responders. Stratified, this resulted in 7 type 1, 7 type 2, and 4 type 3 responders. Recurrences (*n*=17) were positive for CDK4, CDK6, and p-Rb in 29.4%, 64.7%, and 52.9%, respectively, and 52.9% expressed ≤10% p16 protein. From these data, we conclude that 17.6% (3 samples) of the recurrences are eligible as potential responders, 1 as type 1, and 2 as type 2. Metastases (*n*=62) were positive for CDK4, CDK6, and p-Rb in 62.9%, 95.2%, and 53.2%, respectively, and 35.5% expressed ≤10% p16 protein. Taken together, 17.7% (11 samples) of metastases pass as potential responders, including 6 type 1, 4 type 2, and 1 type 3 responder.

Comparison of uterine vs nonuterine leiomyosarcoma showed the following: Uterine tumour samples (*n*=46) were positive for CDK4, CDK6, and p-Rb in 65.2%, 87.2%, and 73.9%, respectively; 30.4% expressed ≤10% p16 protein. Taken together, 21.7% (10 samples) of uterine tumours are eligible as potential responders; four LMS were classified as type 1, five as type 2, and one as type 3. In comparison, nonuterine tumour samples (*n*=113) were positive for CDK4, CDK6, and p-Rb in 54.9%, 86.7%, and 49.6%, respectively, and expressed ≤10% p16 protein in 41.6%. From these, we conclude that nonuterine tumour samples qualify as potential responders in 19.5% (22 samples). Further analysis stratified the nonuterine LMS in 10 type 1, 8 type 2, and 4 type 3 responders.

Given that multiple samples from individual patients were included, patients with one or more positive sample responder profiles were regarded as potential responders. This identified 29 out of 99 (29.3%) patients as potential responders. Furthermore, we investigated the influence of primary tumour site on a potential responder phenotype and found that the percentage of potential responder patients was higher in cases with uterine tumours (10/24, 41.7%) than in retroperitoneal (4/20, 20.0%) or tumours of the extremities and trunk (7/39, 17.9%).

Clinical follow-up data were available for 98 patients. Median overall survival was 4.21 years. Comparison of small tumours (≤5cm) vs large tumours (≥5cm) demonstrated the impact of tumour size on survival time, with a median survival of 9.13 and 3.61, respectively, in both groups. The age at diagnosis was subdivided into ≤60 and >60 years. Median overall survival in those groups was 6.89 compared to 2.49 years. Median overall survival in females (3.65 years) was significantly shorter than that in males (8.82 years). Excluding gynaecologic tumours, patients showed the same results with a change in median overall survival of 3.38 years in female patients.

Median overall survival in the group with uterine leiomyosarcomas was 5.34 years compared to 7.08 years in the group of nonuterine leiomyosarcomas (no statistical significance *p*=0.341). Patients with documented metastasis or recurrences tended to have a shorter median overall survival than those without (no statistical significance *p*=0.057). The potential responder status, Ki-67 index, histological grading, and primary tumour location had no impact on survival, nor did the location of metastasis (pulmonary vs extrapulmonary).

We saw a correlation between the Ki-67 index and histological grading with *r*=0.277 (*p*=0.006). There was no correlation observable between the Ki-67 index and tumour size.

## 4. Discussion

LMS is a highly aggressive tumour with a high morbidity and mortality rate; there is therefore a need for new clinical therapies. We have established a cohort of primary LMS with metastasis and recurrences including a tumour tissue bank. We report a median overall survival of 4.21 years in our LMS cohort, which is in a similar range to that of other studies [13–15]. We observed tumour size as an independent prognostic factor as reported previously [32]. Although histological grading is widely regarded as an independent prognostic marker in sarcomas [33], no difference in survival curves between grading groups was observable in our cohort. However, the lack of this finding is probably attributable to the cohort size. Molecular pathogenesis of LMS remains poorly understood, and previous studies have highlighted the lack of consistent findings, and the complexity of genomic changes within LMS [34]. Our DNA microarray data confirm this finding and document the absence of a distinguishable overall pattern in LMS genetics [1, 18]. In our study, we analysed multiple longitudinal samples from individual patients and were thus able to observe those tumours over time. These data demonstrate heterogeneity within a patient's tumour and elicit the need for repeated testing over the course of disease. We found a higher incidence of aberrations in primary tumours than in recurrences and metastases, and similar findings have been reported for breast cancer [35]. Whole genomic screening for amplifications revealed a recurrent pattern of aberration in LMS. This pattern was characterized by frequent amplifications on chromosomes 12q14.1, 12q15, and 17p12, including genes such as *AGAP2*, *CDK4*, *IL22*, *RAP1B*, *TSPAN31*, *MAP2K4*, and *MYOCD*. These genes are known to be involved in tumorigenesis, cell growth, cell migration, and muscle differentiation [36–39]. One promising candidate found to be amplified was *CDK4* in 2 out of 9 patients. *CDK4* amplification with consecutive overexpression has previously been reported in leiomyosarcoma, although, in contrast to liposarcoma, these findings seem less common [16]. CDK4 is a key regulator in cell cycle progression, and the underlying p16 pathway is therefore frequently altered in various types of cancer [40–43]. Deletion of the 13p region, including the *RB1* locus, is known in a subset of LMS [44], and LMS arising in the setting of hereditary retinoblastoma has been described [45, 46]. The two well-established LMS cell lines, SK-LMS-1 and SK-UT-1, were characterized with regard to the molecules involved in this pathway. On a genetic level, we saw loss of *CDKN2A* in SK-LMS-1 and *RB1* genotype was determined as *RB1* +/+ and *RB1* +/− for SK-LMS-1 and SK-UT-1, respectively. In a study from Coschi et al., SK-LMS-1 is reported as *RB1* +/− and SK-UT-1 as *RB1* −/− [47]. STR profiles confirm the origin of our cell lines, and we therefore attribute these changes to different subclones or heterogeneous *RB1* genotypes. On the protein level, we report that SK-LMS-1 is lacking p16, but Rb and the downstream molecules CDK4, CDK6, and p-Rb are expressed; this is in line with previous reports showing functional Rb protein in SK-LMS-1 [47]. In contrast, no Rb expression was observed in SK-UT-1 combined with expression of p16 protein. These findings confirm data by Francis et al. in the cell line SK-LMS-1 [27].

Loss of p16 has been correlated with a strong growth-inhibitory effect in breast and colon cancer cells, whereas high levels of p16 are considered a surrogate marker of Rb loss, and the combination of both has been shown to correlate with ineffectiveness of palbociclib treatment [48, 49]. Due to the defective p16 pathway in SK-LMS-1, this cell line was analysed for pharmacological inhibition of this pathway by the specific CDK4/CDK6 inhibitor palbociclib. We confirm data of Francis et al. which show that Rb phosphorylation was reduced in a dose-dependent manner after palbociclib treatment of the cell line; in line with this observation, we consistently saw a decrease of cell growth rate in our cell counting experiments [27]. As CDK4/6 plays an important role in cycle progression, we predicted that, in this setting, growth inhibition of the LMS cell should lead to an exclusive block at the G_1_ phase checkpoint. We confirmed this to be the case and observed selective G_1_ phase increase with a consequent decrease of cells in the S and G_2_ phase. Based on the mechanism of action of palbociclib, the anticancer activity is mainly achieved by cell cycle arrest, and apoptosis does not appear to be the key event [31, 48]. Consistent with this finding, we saw a small increase in the sub-G_1_ phase only, and no apoptotic activity was seen, as shown by the absence of an increase in cleaved caspase-3 in immunohistochemistry. Furthermore, we confirm that palbociclib-treated cells undergo morphological changes, such as formation of multinuclear cells, which has been described in the context of senescence [50]. This finding is in line with previous studies, which have analysed this pathway in the LMS cell line SK-LMS-1 and chordomas, breast cancer, and other sarcomas [21, 26, 27, 31, 48]. Our study confirms and extends knowledge of published data which already showed a positive effect of palbociclib leiomyosarcoma cell lines and a leiomyosarcoma sample xenograft transplanted in mice [26, 27]. No inhibitory effect was seen for SK-UT-1, confirming the previously stated requirements for effective palbociclib treatment.

Finally, we analysed the expression of p16, CDK4, CDK6, and p-Rb in our samples, in order to detect a potential responder phenotype for treatment with palbociclib and subsequently compared those findings with cell lines that are known to respond to treatment. We categorized the patient samples in groups based on their probability of being treatment responders or nonresponders. The responder group was characterized by p16 negativity and the presence of p-Rb and either CDK4 or CDK6. If not all of those criteria could be met, the sample was categorized as nonresponder, which, by contrast, was characterized by either p16 positivity or absence of CDK4 and CDK6 or p-Rb [31]. Out of all the leiomyosarcoma patients, 65.7% were positive for both CDK4 and CDK6. Nevertheless, nearly one-third of patients analysed (31.3%) had p-Rb protein depletion, and these patients therefore have to be considered as unsuitable for potential palbociclib treatment. Other tumour samples showed expression of CDK4, CDK6, and p-Rb protein but also p16 expression, which is associated with ineffectiveness of palbociclib, and these patients were therefore regarded as potential nonresponders.

Ultimately, we dissected a number of patients (29.3%) who met all the criteria for a functional p16 axis, making these patients potential responders for treatment with cell cycle kinase inhibitors such as palbociclib. Our large cohort allowed us to analyse paired tumour samples from individual patients collected over a period of up to 141 months. We found that expression of the various constituents of the p16 pathway may vary over time. This highlights the importance of repeatedly evaluating the protein profile when searching for possible treatment options such as palbociclib. The latter received FDA approval for treatment of metastatic breast cancer in February 2016 [51] and has been studied in various clinical trials researching its effectiveness in various other cancer types, such as mantle cell lymphoma, multiple myeloma, germ cell tumours, nonsmall cell lung cancer, and dedifferentiated liposarcoma in Phase I-II. Those studies demonstrated favourable effects on the progression-free survival, and treatment showed only moderate side effects [20, 23–25, 52, 53]. Therefore, our data may be considered as the starting point for a new therapeutic regimen such as palbociclib in LMS.

## 5. Conclusion

Treatment of leiomyosarcoma (LMS) is challenging, and little progress regarding a targeted therapy has been achieved so far.

In this study, we have identified CDK4 amplification as a recurrent alteration in LMS in a genome-wide approach. To analyse the role of CDK4 in LMS, we studied the LMS cell lines SK-UT-1 and SK-LMS-1. Both lines express CDK4 and, in addition, SK-LMS-1 was strongly positive for CDK6. The p16 protein was absent in SK-LMS-1 but not in SK-UT-1. Therefore, in SK-LMS-1, the absence of p16 results in a universal activation of the p16 pathway. We further studied whether this pathway is targetable in these cell lines by a CDK4/CDK6 inhibitor. We confirm that in SK-LMS-1, inhibition with palbociclib led to a strong decrease in of Phospho-Rb (Ser780) protein levels, a decreased cell proliferation, and G_0_/G_1_-phase arrest. To compare our *in vitro* findings with patient tissue samples, a p16, CDK4, CDK6, and p-Rb immunohistochemical staining assay for profiling of a large LMS cohort (*n*=99 patients with 159 samples) was performed assigning a potential responder phenotype to each patient, which we identified in 29 of 99 (29.3%) patients. These data show that CDK4/6 inhibitors may offer a new option for targeted therapy in a subset of LMS patients.

## Figures and Tables

**Table 1:** Recurrent high copy gains in leiomyosarcoma samples analysed by OncoScan. Cytoband location as well as DNA base range according to human genome assembly *GRCh37* is annotated. The event frequency and lists of genes in the corresponding region are outlined.

Cytoband	Region	Length (bp)	Frequency (%)	Genes	Gene symbols
12q14.1	chr12:58.125.215–58.255.151	129936	27.8	10	*AGAP2, TSPAN31, CDK4, MARCH9, CYP27B1, METTL1, METTL21B, TSFM, AVIL, CTDSP2*
12q15	chr12:68.593.003–69.052.981	459978	27.8	4	*IL26, IL22, MDM1, RAP1B*
17p12	chr17:12.000.608–12.087.625	87017	38.9	1	*MAP2K4*
17p12	chr17:12.105.562–12.579.457	473895	38.9	1	*MYOCD*

## Data Availability

All the data presented in this study are available upon reasonable request by contacting the corresponding author.

## Supplementary Materials

Supplementary Materials Supplementary Figure S1: Western Blot (A) Western blot analysis of LMS cell lines SK-LMS-1 and SK-UT-1 for p16 pathway proteins Rb, CDK6, CDK4, and p16 compared to HeLa cells. *β*-tubulin was used as loading control. (B) Inhibition assay of SK-LMS-1 over 24 and 48 hours with palbociclib concentrations ranging from 100–2000 nmol/l. The second row from above shows a concentration-dependent decrease in p-Rb (Ser780). ERK2 is shown as loading control. Supplementary Figure S2: FACS and cell-counting (A) flow cytometric cell cycle analysis for SK-LMS-1 after 24 and 48 hours of palbociclib inhibition. ^*∗*^=*p* ≤ 0.05, ^*∗∗*^=*p* ≤ 0.01, and ^*∗∗∗∗*^=*p* ≤ 0.0001. (B) Cell counting graph of SK-LMS-1. Incubation with 100 and 1000 nmol/l concentrations of palbociclib for up to 3 days, followed by automated cell counting every 24 hours. Supplementary Figure S3: Microscopic findings of SK-LMS-1 after palbociclib inhibition. The left side shows the untreated sample for comparison purposes. (A) Treated cells show a decrease in Ki-67 as expression of growth inhibition. (B) Cleaved caspase-3 staining demonstrates no apoptotic activity. (C) May–Grünwald–Giemsa (MGG) staining of cells directly cultivated on microscopic slides. Treated cells show formation of multinuclear cells. Bars: 100 *µ*m. Supplementary Table S1: Correlation of gene status and protein expression of members of the p16 pathway. For each sample, OncoScan calls of high copy gains and biallelic losses are compared to the percentage of positively stained cells by immunohistochemistry. Abbreviations: P, primary tumour; M, metastasis; R, recurrence; T, tumour type; HCG, high copy gain; BL, biallelic loss; IHC, immunohistochemistry. Supplementary Table S2: Sample overview of Affymetrix OncoScan cohort. Uterine-derived tumours are marked with asterisks. Supplementary Table S3: Clinical data table. Age, gender, and primary tumour grading for each patient are given. Available data on primary tumour size (in centimetres) and documented metastasis are outlined alongside life status and observed survival. Abbreviations: f, female; m, male; 0, alive; 1, dead; OS, overall survival. Supplementary Table S4: STR profiles of leiomyosarcoma cell lines. Comparison of STR profiles from ATCC and ExPasY database to cell lines used in our in vitro experiments. Matching STRs are highlighted by green background.
