# Supplementary material for: Analysis of the CDK4/6 Cell Cycle Pathway in Leiomyosarcomas as a Potential Target for Inhibition by Palbociclib
Source: Sarcoma. 2019 Jan 21;2019:3914232. doi: 10.1155/2019/3914232 (PMC6360577; doi:10.1155/2019/3914232)
Supplement: Supplementary Materials — Supplementary Figure S1: Western Blot (A) Western blot analysis of LMS cell lines SK-LMS-1 and SK-UT-1 for p16 pathway proteins Rb, CDK6, CDK4, and p16 compared to HeLa cells. β-tubulin was used as loading control. (B) Inhibition assay of SK-LMS-1 over 24 and 48 hours with palbociclib concentrations ranging from 100–2000 nmol/l. The second row from above shows a concentration-dependent decrease in p-Rb (Ser780). ERK2 is shown as loading control. Supplementary Figure S2: FACS and cell-counting (A) flow cytometric cell cycle analysis for SK-LMS-1 after 24 and 48 hours of palbociclib inhibition. ∗=p ≤ 0.05, ∗∗=p ≤ 0.01, and ∗∗∗∗=p ≤ 0.0001. (B) Cell counting graph of SK-LMS-1. Incubation with 100 and 1000 nmol/l concentrations of palbociclib for up to 3 days, followed by automated cell counting every 24 hours. Supplementary Figure S3: Microscopic findings of SK-LMS-1 after palbociclib inhibition. The left side shows the untreated sample for comparison purposes. (A) Treated cells show a decrease in Ki-67 as expression of growth inhibition. (B) Cleaved caspase-3 staining demonstrates no apoptotic activity. (C) May–Grünwald–Giemsa (MGG) staining of cells directly cultivated on microscopic slides. Treated cells show formation of multinuclear cells. Bars: 100 µm. Supplementary Table S1: Correlation of gene status and protein expression of members of the p16 pathway. For each sample, OncoScan calls of high copy gains and biallelic losses are compared to the percentage of positively stained cells by immunohistochemistry. Abbreviations: P, primary tumour; M, metastasis; R, recurrence; T, tumour type; HCG, high copy gain; BL, biallelic loss; IHC, immunohistochemistry. Supplementary Table S2: Sample overview of Affymetrix OncoScan cohort. Uterine-derived tumours are marked with asterisks. Supplementary Table S3: Clinical data table. Age, gender, and primary tumour grading for each patient are given. Available data on primary tumour size (in centimetres) and documented metastasis [file 3914232.f1.pptx]

## Slide 1
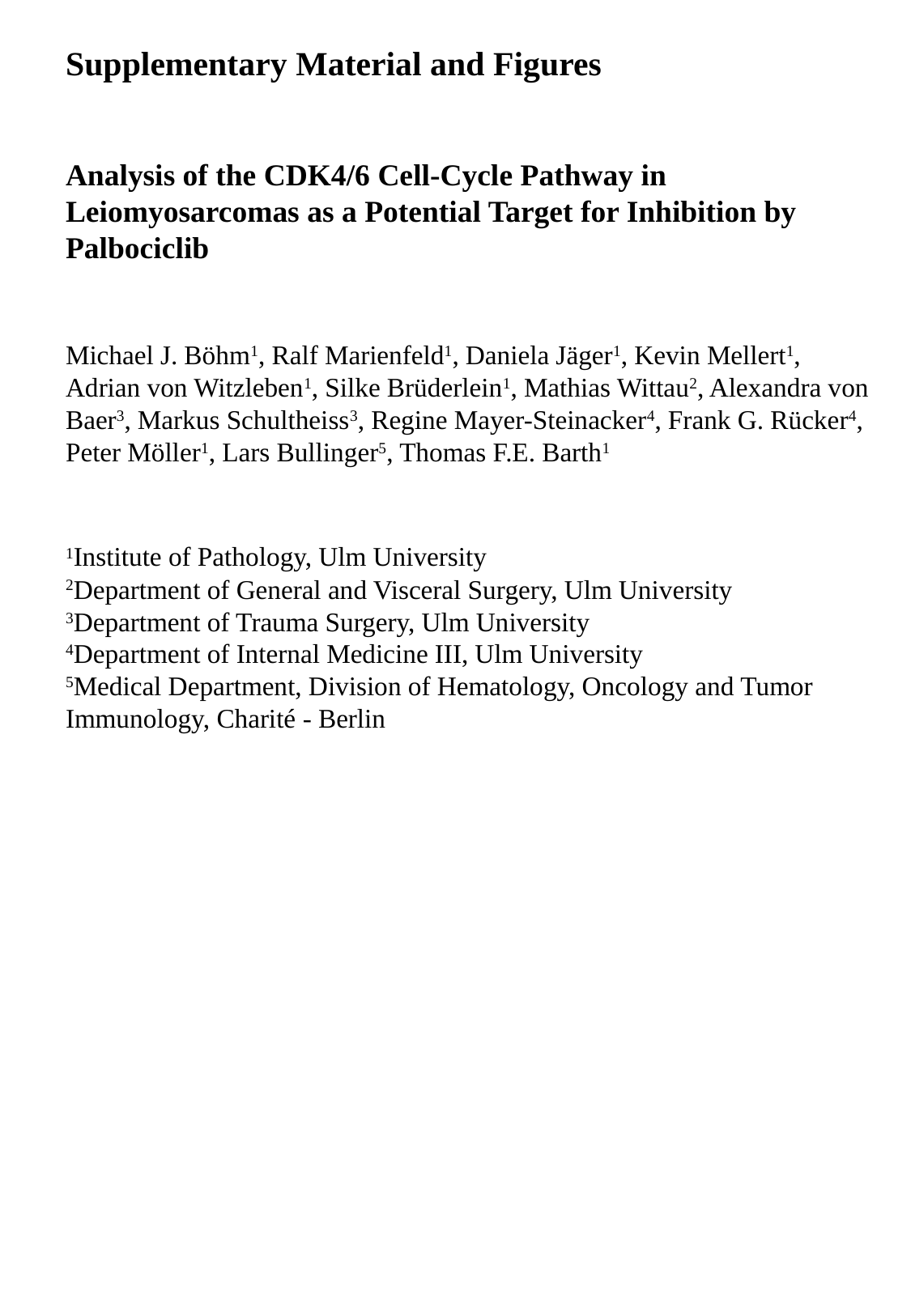

Supplementary Material and Figures
Analysis of the CDK4/6 Cell-Cycle Pathway in Leiomyosarcomas as a Potential Target for Inhibition by Palbociclib
Michael J. Böhm1, Ralf Marienfeld1, Daniela Jäger1, Kevin Mellert1, Adrian von Witzleben1, Silke Brüderlein1, Mathias Wittau2, Alexandra von Baer3, Markus Schultheiss3, Regine Mayer-Steinacker4, Frank G. Rücker4, Peter Möller1, Lars Bullinger5, Thomas F.E. Barth1
1Institute of Pathology, Ulm University
2Department of General and Visceral Surgery, Ulm University
3Department of Trauma Surgery, Ulm University
4Department of Internal Medicine III, Ulm University
5Medical Department, Division of Hematology, Oncology and Tumor Immunology, Charité - Berlin

## Slide 2
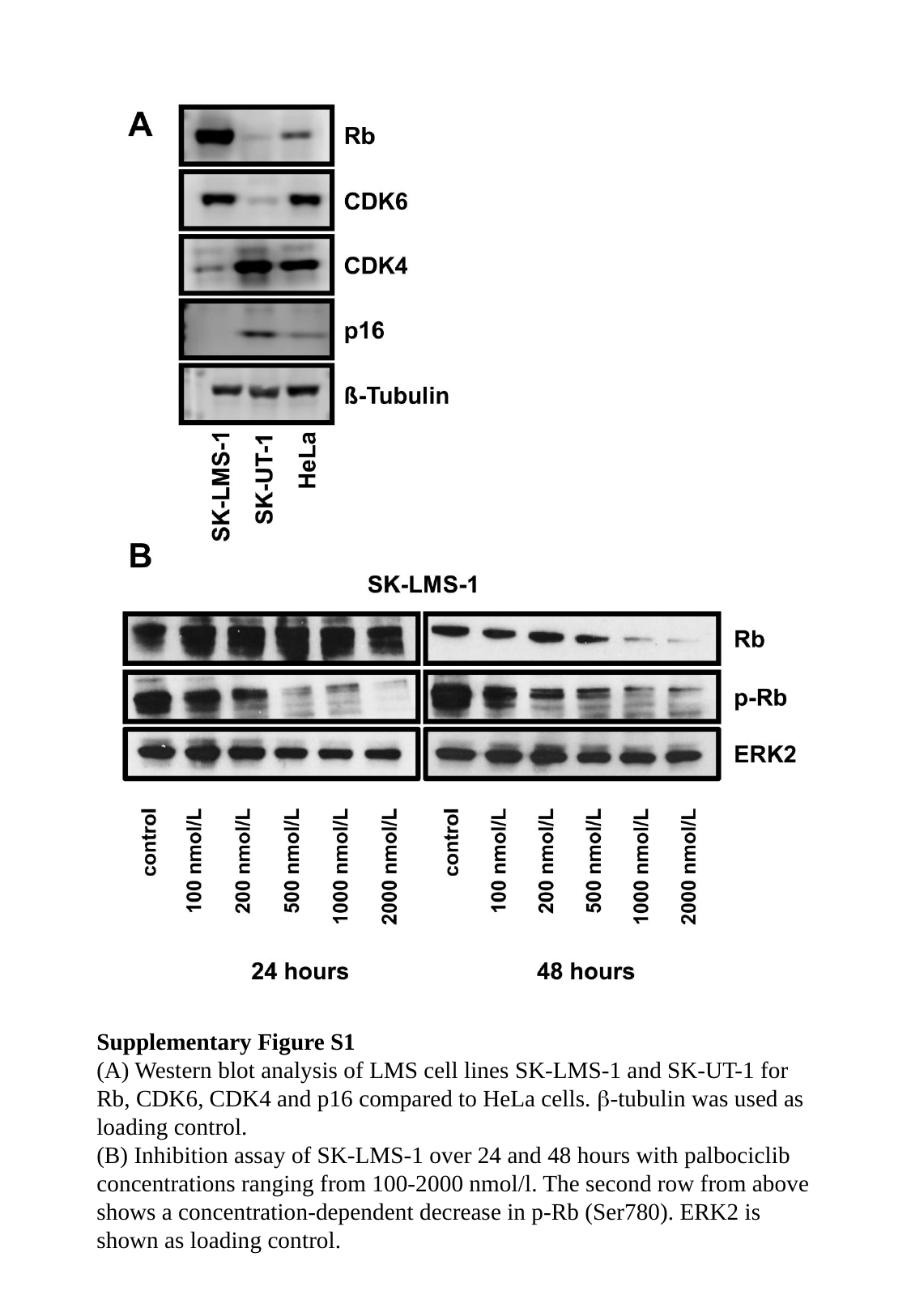

Supplementary Figure S1
(A) Western blot analysis of LMS cell lines SK-LMS-1 and SK-UT-1 for Rb, CDK6, CDK4 and p16 compared to HeLa cells. β-tubulin was used as loading control.
(B) Inhibition assay of SK-LMS-1 over 24 and 48 hours with palbociclib concentrations ranging from 100-2000 nmol/l. The second row from above shows a concentration-dependent decrease in p-Rb (Ser780). ERK2 is shown as loading control.

## Slide 3
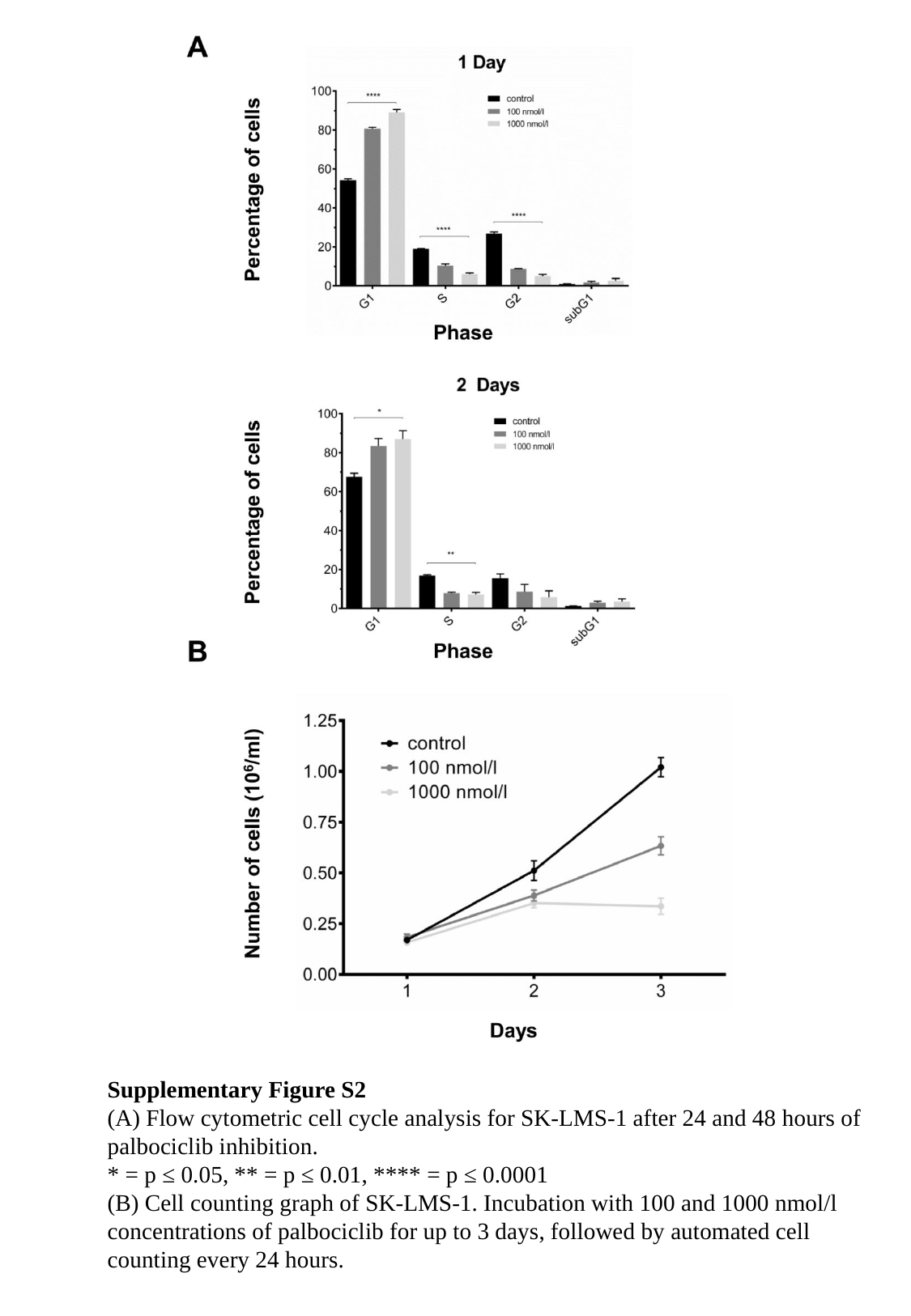

Supplementary Figure S2
(A) Flow cytometric cell cycle analysis for SK-LMS-1 after 24 and 48 hours of palbociclib inhibition.
* = p ≤ 0.05, ** = p ≤ 0.01, **** = p ≤ 0.0001
(B) Cell counting graph of SK-LMS-1. Incubation with 100 and 1000 nmol/l concentrations of palbociclib for up to 3 days, followed by automated cell counting every 24 hours.

## Slide 4
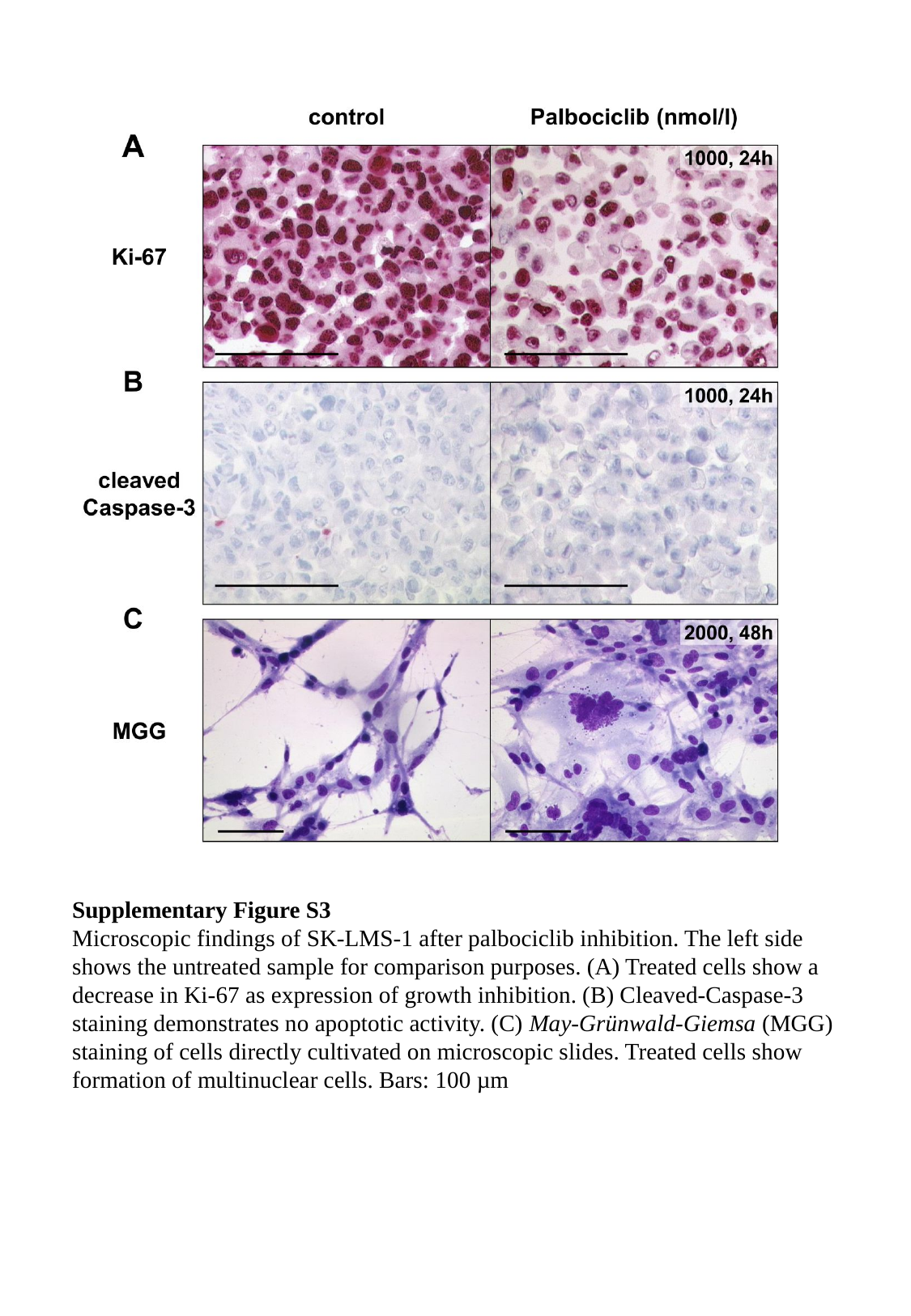

Supplementary Figure S3
Microscopic findings of SK-LMS-1 after palbociclib inhibition. The left side shows the untreated sample for comparison purposes. (A) Treated cells show a decrease in Ki-67 as expression of growth inhibition. (B) Cleaved-Caspase-3 staining demonstrates no apoptotic activity. (C) May-Grünwald-Giemsa (MGG) staining of cells directly cultivated on microscopic slides. Treated cells show formation of multinuclear cells. Bars: 100 µm

## Slide 5
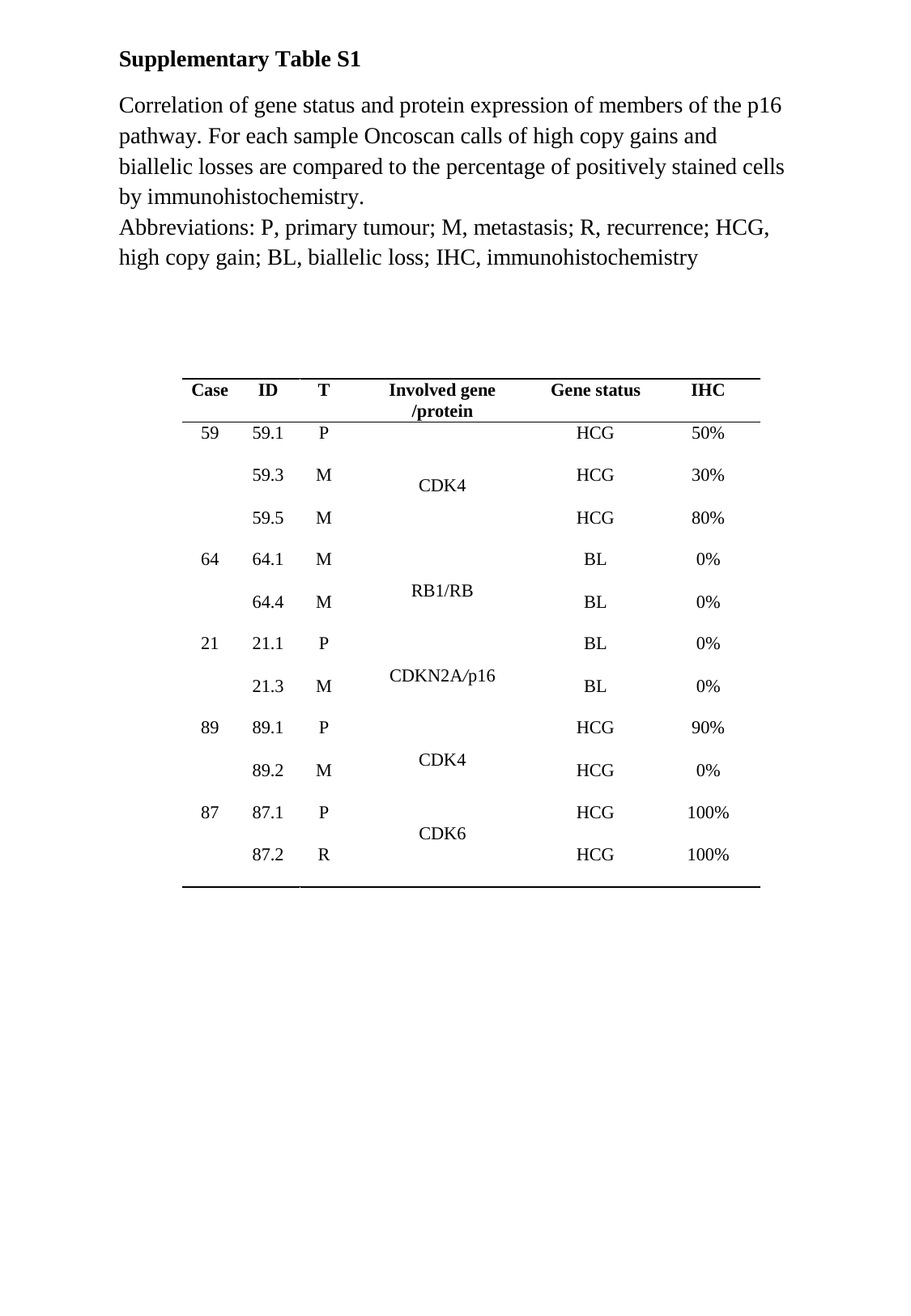

## Slide 6
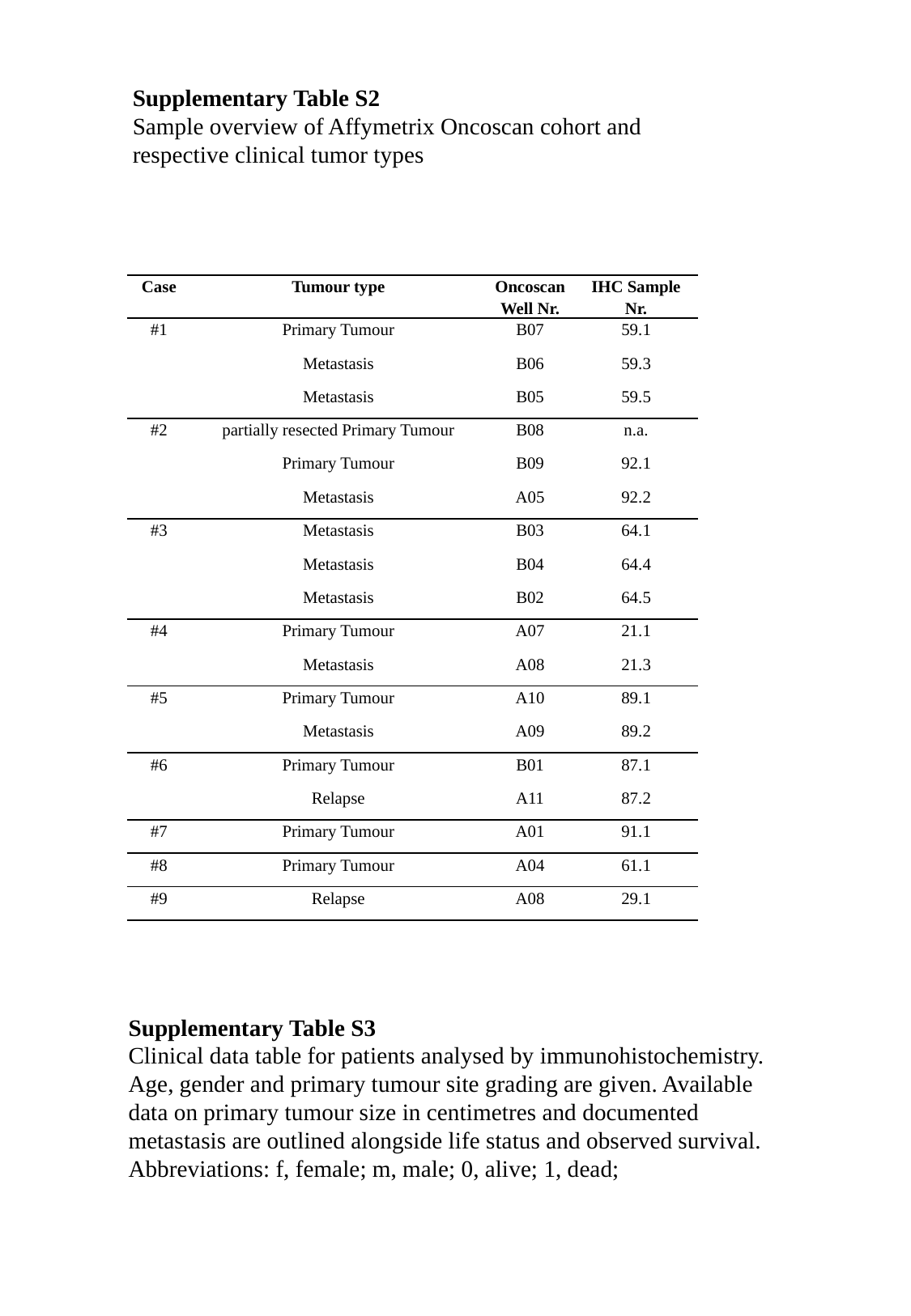

Supplementary Table S2
Sample overview of Affymetrix Oncoscan cohort and respective clinical tumor types
| Case | Tumour type | Oncoscan Well Nr. | IHC Sample Nr. |
| --- | --- | --- | --- |
| #1 | Primary Tumour | B07 | 59.1 |
| | Metastasis | B06 | 59.3 |
| | Metastasis | B05 | 59.5 |
| #2 | partially resected Primary Tumour | B08 | n.a. |
| | Primary Tumour | B09 | 92.1 |
| | Metastasis | A05 | 92.2 |
| #3 | Metastasis | B03 | 64.1 |
| | Metastasis | B04 | 64.4 |
| | Metastasis | B02 | 64.5 |
| #4 | Primary Tumour | A07 | 21.1 |
| | Metastasis | A08 | 21.3 |
| #5 | Primary Tumour | A10 | 89.1 |
| | Metastasis | A09 | 89.2 |
| #6 | Primary Tumour | B01 | 87.1 |
| | Relapse | A11 | 87.2 |
| #7 | Primary Tumour | A01 | 91.1 |
| #8 | Primary Tumour | A04 | 61.1 |
| #9 | Relapse | A08 | 29.1 |
Supplementary Table S3
Clinical data table for patients analysed by immunohistochemistry. Age, gender and primary tumour site grading are given. Available data on primary tumour size in centimetres and documented metastasis are outlined alongside life status and observed survival. Abbreviations: f, female; m, male; 0, alive; 1, dead;

## Slide 7
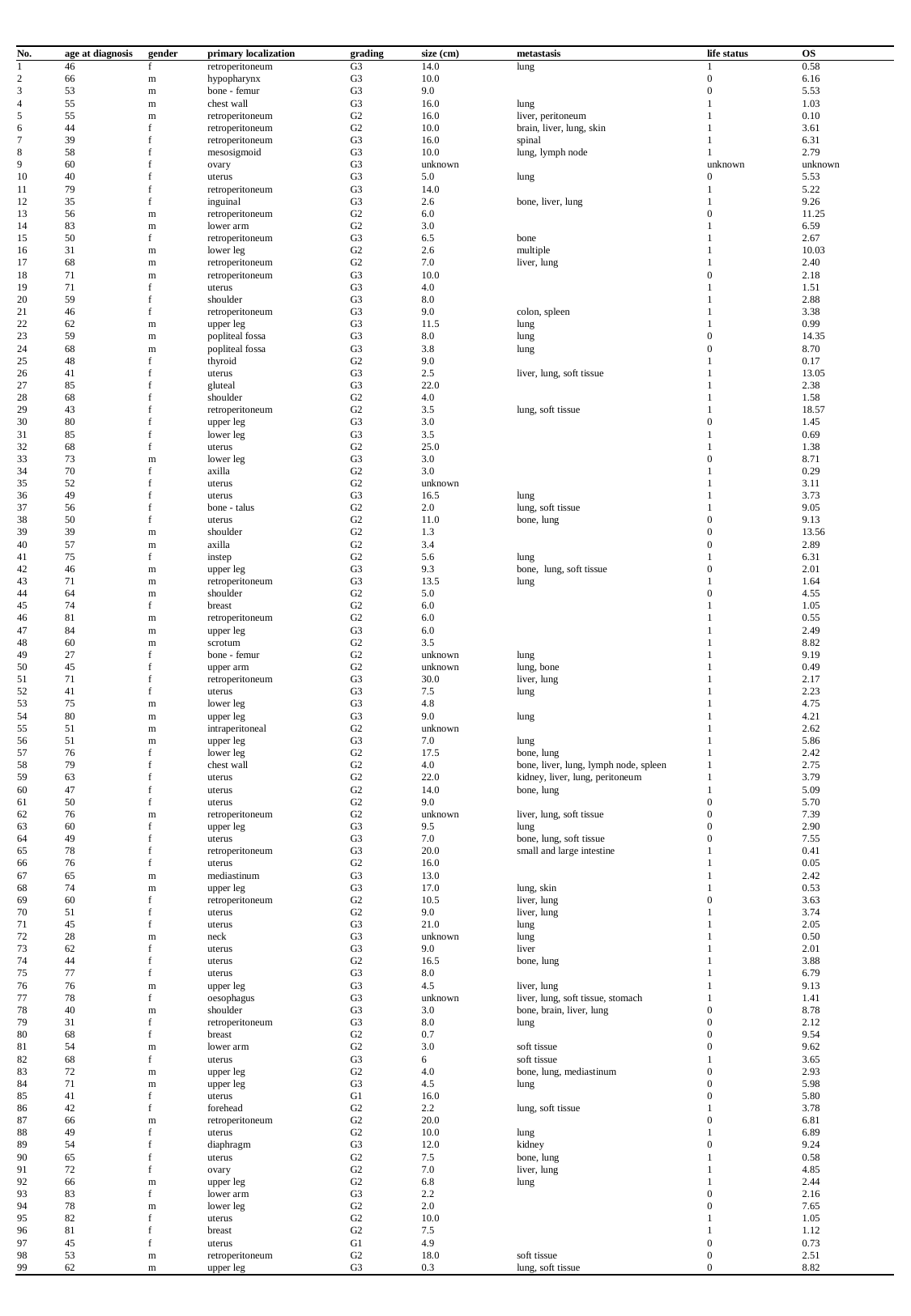

## Slide 8
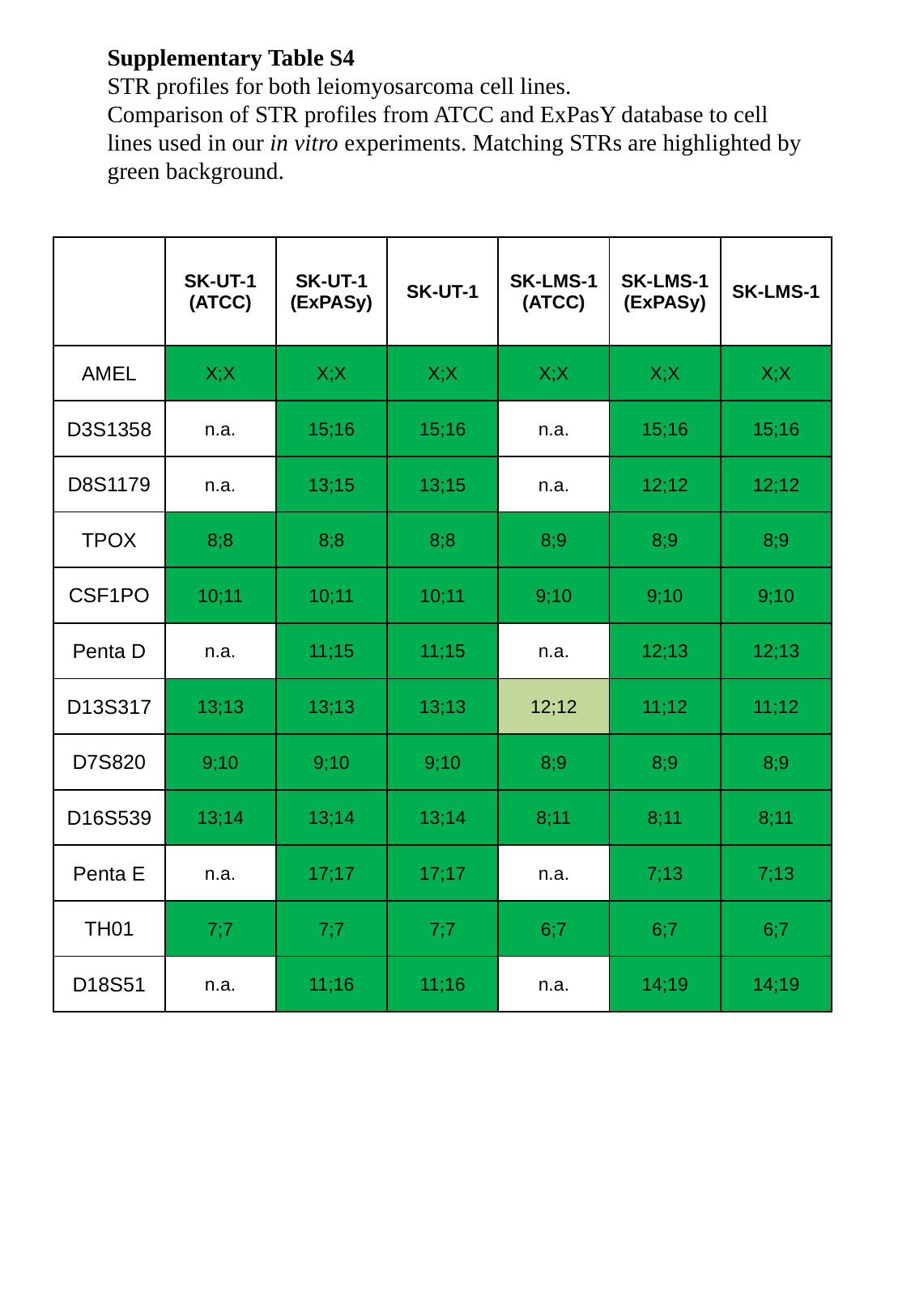

Supplementary Table S4
STR profiles for both leiomyosarcoma cell lines.
Comparison of STR profiles from ATCC and ExPasY database to cell lines used in our in vitro experiments. Matching STRs are highlighted by green background.
| | SK-UT-1 (ATCC) | SK-UT-1 (ExPASy) | SK-UT-1 | SK-LMS-1 (ATCC) | SK-LMS-1 (ExPASy) | SK-LMS-1 |
| --- | --- | --- | --- | --- | --- | --- |
| AMEL | X;X | X;X | X;X | X;X | X;X | X;X |
| D3S1358 | n.a. | 15;16 | 15;16 | n.a. | 15;16 | 15;16 |
| D8S1179 | n.a. | 13;15 | 13;15 | n.a. | 12;12 | 12;12 |
| TPOX | 8;8 | 8;8 | 8;8 | 8;9 | 8;9 | 8;9 |
| CSF1PO | 10;11 | 10;11 | 10;11 | 9;10 | 9;10 | 9;10 |
| Penta D | n.a. | 11;15 | 11;15 | n.a. | 12;13 | 12;13 |
| D13S317 | 13;13 | 13;13 | 13;13 | 12;12 | 11;12 | 11;12 |
| D7S820 | 9;10 | 9;10 | 9;10 | 8;9 | 8;9 | 8;9 |
| D16S539 | 13;14 | 13;14 | 13;14 | 8;11 | 8;11 | 8;11 |
| Penta E | n.a. | 17;17 | 17;17 | n.a. | 7;13 | 7;13 |
| TH01 | 7;7 | 7;7 | 7;7 | 6;7 | 6;7 | 6;7 |
| D18S51 | n.a. | 11;16 | 11;16 | n.a. | 14;19 | 14;19 |
